# Study on Corrosion Behavior and Mechanism of Ultrahigh-Strength Hot-Stamping Steel Based on Traditional and Compact Strip-Production Processes

**DOI:** 10.3390/ma16083064

**Published:** 2023-04-13

**Authors:** Guoqiang Ma, Yimian Chen, Shuize Wang, Honghui Wu, Junheng Gao, Guilin Wu, Xinping Mao

**Affiliations:** 1 Innovation Research Institute for Carbon Neutrality, University of Science and Technology Beijing, Beijing 100083, China; guomacn@ustb.edu.cn (G.M.);; 2 Yangjiang Branch, Guangdong Laboratory for Materials Science and Technology (Yangjiang Advanced Alloys Laboratory), Yangjiang 529500, China

**Keywords:** compact strip production, hot-stamping steel, martensite, corrosion, inclusion

## Abstract

Hot-stamping steel is a type of high-strength steel that is mainly used in key safety components such as the front and rear bumpers, A-pillars, and B-pillars of vehicles. There are two methods of producing hot-stamping steel, i.e., the traditional process and the near net shape of compact strip production (CSP) process. To assess the potential risks of producing hot-stamping steel using CSP, the microstructure and mechanical properties, and especially the corrosion behavior were focused on between the traditional and CSP processes. The original microstructure of hot-stamping steel produced by the traditional process and the CSP process is different. After quenching, the microstructures transform into full martensite, and their mechanical properties meet the 1500 MPa grade. Corrosion tests showed that the faster the quenching speeds, the smaller the corrosion rate of the steel. The corrosion current density changes from 15 to 8.6 μA·cm^−2^. The corrosion resistance of hot-stamping steel produced by the CSP process is slightly better than that of traditional processes, mainly since the inclusion size and distribution density of CSP-produced steel were both smaller than those of the traditional process. The reduction of inclusions reduces the number of corrosion sites and improves the corrosion resistance of steel.

## 1. Introduction

Hot-stamping steel is a type of ultrahigh-strength steel that is specially designed for the production of high-strength and high-plasticity components, and it possesses excellent thermal plasticity, workability, and formability [1,2,3,4,5,6]. Due to its exceptional forming performance, hot-stamping steel can be plastically deformed at high temperatures, resulting in precise product shapes and sizes. In addition, it has good mechanical properties, wear resistance, strength, toughness, and corrosion resistance, making it a suitable material for use in various fields such as automobiles, machinery, aviation, and aerospace. One of the most commonly used hot-stamping steels is 22MnB5 (1500 MPa grade), which is used for automotive safety components such as front and rear bumpers, A-pillars, and B-pillars [7,8,9,10,11,12,13,14,15].

The traditional production methods of ultrahigh-strength steel usually include hot rolling, pickling, cold rolling, and annealing. Such a process has long product lines, high energy consumption, a long production cycle, and high production cost. In recent years, new production methods have been developed for short processes, such as the compact strip-production (CSP) process, which belongs to the method of near-net shape [16,17,18,19,20,21,22,23]. Thin slabs are hot rolled and coiled directly, and cold rolling and annealing processes are canceled in the CSP process. The CSP process has great potential in terms of energy saving and economic benefits, which can greatly reduce the production cost of materials and parts [16,18,24]. However, the CSP process has several potential disadvantages or challenges that need to be addressed. For example, it may not be as suitable for the products of thicker plates or more complex shapes. The high production speed of CSP can make it challenging to maintain the consistency of the microstructure and product quality, and there may be a higher risk of defects or surface imperfections compared to traditional production methods such as the changes in the type and distribution of inclusions and precipitations due to the difference in the process [16,19,20,25,26,27].

To assess the potential risks of producing hot-stamping steel using CSP, multiple studies have been performed to investigate the microstructure, mechanical properties, forming behavior, and hydrogen embrittleness, as well as the optimization of the CSP process parameters for producing hot-stamping steel with desired properties. Jin et al. [24] reported a more competitive press-hardening steel design based on C-Mn steel addition with Cr and B elements produced by the CSP process, which can induce good comprehensive performance and economy. Zhou et al. [28] found that the original microstructure has an effect on the microstructure and mechanical properties of the press-hardening steel produced by the CSP process. The austenite grains after austenitization are fine and uniform when the original structure is ferrite and pearlite, while the austenite grains after austenitization are coarse when the original structure is martensite. In addition, the strength after quenching is inverse to the strength of the original steel. Chen et al. [29,30] showed that the inclusions of hot-stamping steels produced by CSP are mainly spherical Al-Ca-O and CaS, while the inclusions in the traditional hot-stamping steels are TiN + Al_2_O_3_ + MnS with sharp edges and corners. The hydrogen embrittlement resistance of hot-stamping steels produced by CSP is superior to that of the traditional hot-stamping steels due to the influence of composition, shape, and distribution of inclusions.

The corrosion resistance of steel can also have an effect on the performance and lifespan of the automotive components since the parts must endure harsh conditions involving high temperatures and humidity. However, there is, to the best knowledge of the authors, no extensive research on the effect of manufacturing processes on the corrosion resistance of hot-stamping steels. It is well known that the chemical composition of the steel matrix, the microstructure, the composition, and the size distribution of the inclusions, the second phase, and the texture will have a great influence on the corrosion resistance, while the CSP process will change the influencing factors mentioned above compared to the traditional process. Therefore, the hot-stamping steel produced by the CSP and the traditional processes were chosen in this study. The mechanical property of steel sheets before and after heat treatment for the two production methods was tested, and the microstructures corresponding were analyzed. The corrosion behavior and mechanism of the steel sheet after heat treatment were investigated comprehensively.

## 2. Materials and Methods

The starting steel sheets were 1500 MPa grade hot-stamping steel taken from Hunan Valin Lianyuan Steel and produced by CSP and traditional processes. In the CSP process, the finisher entry and delivery temperature during hot rolling is controlled at 1080 °C and 890 °C, and the coiling temperature is controlled at 600 °C. In the traditional process, the slab discharging temperature is controlled at 1230 °C, the finisher delivery temperature is controlled at 890 °C, and the coiling temperature is controlled at 625 °C. In addition, the sheets produced by the traditional process were cold rolled and annealed at 680 °C for 10 h. The final thicknesses of steel sheets are 2 mm and 1.8 mm for the CSP and traditional processes, respectively. All samples were cut near the typical 1/4 width of the steel sheets. Optical emission spectrometry was used to test the chemical composition of the experimental steels, and the chemical composition is shown in Table 1.

The Ac1 and Ac3 temperatures for the present steels are about 720 °C and 850 °C, respectively, calculated from Andrew’s relationship [31]. The heat treatment temperature of 920 °C was thus selected for the present work based on Ac3 to complete austenitizing. The heat treatment time (T) of 4 min was selected based on an empirical formula:

T = K × D(1)

where D is the thickness of the sample in millimeters and K is the heating coefficient taken to be 2 min/mm. After austenitizing, the samples were quenched with different cooling rates by quenching in oil, water, and 10 wt.% NaCl solution, respectively.

The original microstructure and the microstructure after quenching were observed by scanning electron microscope (SEM) and electron backscatter diffraction (EBSD). Inclusions in the steels were analyzed by a backscattered electron (BSE). The sizes of the inclusions were measured using the software Nano Measurer 1.2.5. The compositions of these inclusions were identified by energy disperse spectroscopy (EDS). There are five regions, each with a field size of approximately 0.62 mm^2^ (950 μm × 650 μm), for a total area greater than 3 mm^2^, that were analyzed using statistical methods. The inclusions sized less than 0.5 um were not included. The microstructural analysis was performed on the longitudinal section, which encompasses both the rolling direction (RD) and the normal direction (ND).

The MTS Exceed E45 electronic universal test system was used to conduct the tensile test, and a mechanical extensometer with a 50 mm gauge length was used to collect real-time strain during the test. Dog-bone-shaped tensile samples were cut from the sheets, with a gauge length of 50 mm, width of 12.5 mm, and thickness equal to that of the original sheets. Prior to the test, the gauge section’s thickness and width were measured using a vernier caliper. The test was conducted at a constant strain rate of 1 mm/min. To ensure accurate results, at least three samples were prepared for the tensile test. If the stress-strain curve exhibited a yield point, the stress corresponding to the lower yield point was taken as the yield strength. Otherwise, the stress corresponding to a plastic strain of 0.2% was used as the yield strength [32].

Electrochemical samples were cut into circular pieces with a diameter of 10 mm. The circular shape was designed to avoid the additional accelerated corrosion effect caused by the sharp corners of a square sample. The circular pieces were connected to copper wires using a soldering iron and encapsulated with epoxy resin and a curing agent in a 5:1 ratio, leaving a working surface of 0.785 cm^2^. During electrochemical corrosion testing, specimens were exposed to a 3.5% NaCl solution at room temperature. To conduct the electrochemical measurements, a conventional three-electrode cell system was used in a CS350 electrochemical workstation, with the sample as the working electrode, a saturated calomel electrode (SCE) as the reference electrode, and a 1 cm^2^ Pt electrode as the counter electrode. The potentiodynamic polarization curve was tested at a scan rate of 1 mV/s with a scan ranging from −1.0 V (SCE) to 0.5 V (SCE), and the data were fitted using Cview 2.6 software [33]. The EIS tests were carried out when samples are stabilized in the test solution within a frequency range of 100 kHz to 10 mHz and a sinusoidal perturbation with 10 mV amplitude, and then the data were analyzed by the software Zview 3.0a.

## 3. Results and Discussion

**Figure 1:**
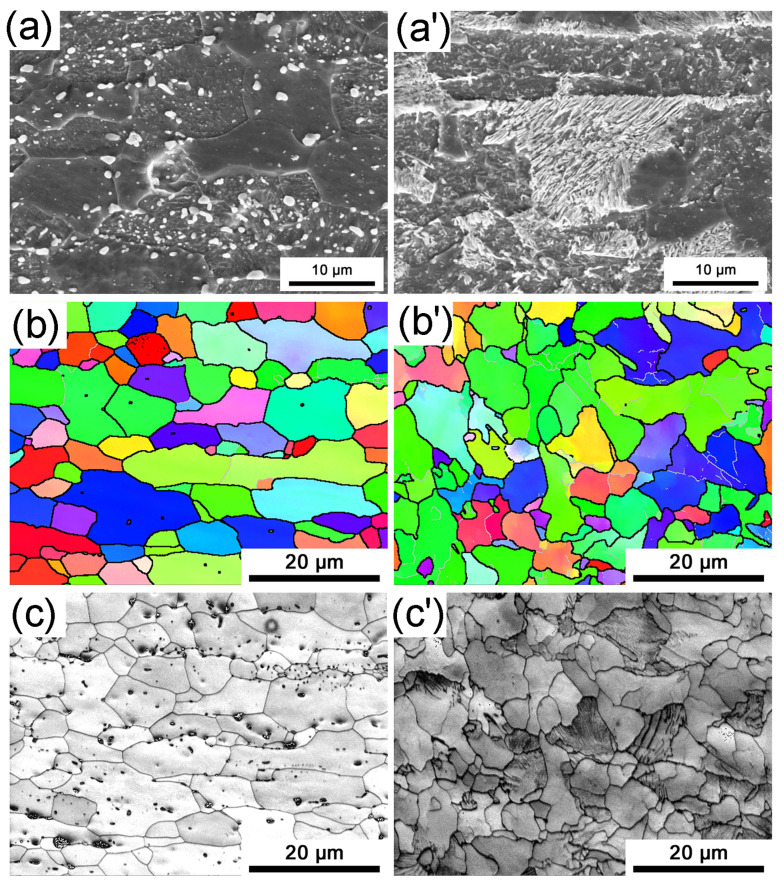
The original microstructure of hot-stamping steel for two processes: SEM images of the (**a**) traditional process and (**a’**) CSP process, EBSD inverse pole figure (IPF) maps of the (**b**) traditional process and (**b’**) CSP process, and corresponding band contrast of the (**c**) traditional process and (**c’**) CSP process.

The original microstructures of hot-stamping steel produced by the traditional and CSP processes are shown in Figure 1. In the traditional process, equiaxed polygonal ferrite grains were observed, with spheroidized carbides/pearlites dispersed within the grain boundaries and interiors. The carbides/pearlites and ferrite grains are alternatively distributed in lamellar structures along the deformation direction. During cold rolling, the carbides/pearlites in the traditional process are fractured, followed by a long isothermal annealing process, during which the cold-deformed ferrite grains undergo recrystallization to form equiaxed polygonal grains, while the fragmented carbides undergo spheroidization. In the CSP process, where there are no cold rolling and annealing processes, the microstructure is a typical room-temperature structure of hypoeutectoid steel, consisting of ferrite and pearlite. The pearlite lamellar spacing is about 0.1–0.15 μm. The typical band structure formed by hot rolling is retained in the matrix. The original grain size of the hot-stamping steel produced by the traditional process is 5.6 μm, while that of the hot-stamping steel produced by the CSP process is 3.9 μm.

**Figure 2:**
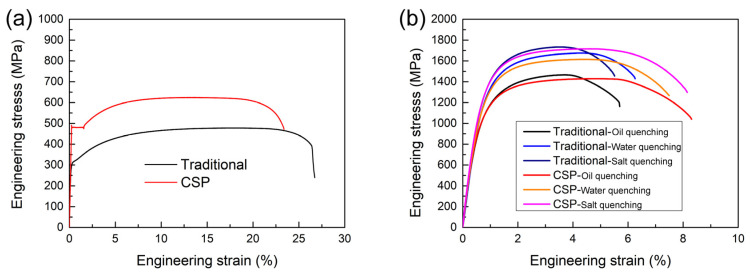
Mechanical properties of traditional and CSP processes produced steels: (**a**) engineering stress–strain curve of the original samples and (**b**) engineering stress–strain curve after quenching by a different quenching medium.

The mechanical properties of hot-stamping steel before and after quenching by traditional and CSP processes are shown in Figure 2. The tensile strength of steel produced by the traditional process is the lowest, at 473 MPa, with an elongation of 26%. This is mainly due to the traditional process steel undergoing an annealing process at 680 °C for 10 h. The CSP process samples have a higher tensile strength of 630 MPa and a lower elongation of 23%. The strength and plasticity of the original sheets are inversely related. The mechanical properties of the samples after quenching are greatly improved, and the steel plates produced by both the traditional process and the CSP process meet the requirements of 1500 MPa tensile strength, 1000 MPa yield strength, and 5% elongation after heat treatment. In addition, the strength of the sheets increases with the increase of the quenching cooling rate. The mechanical property parameters after quenching are tabulated in Table 2. 

**Figure 3:**
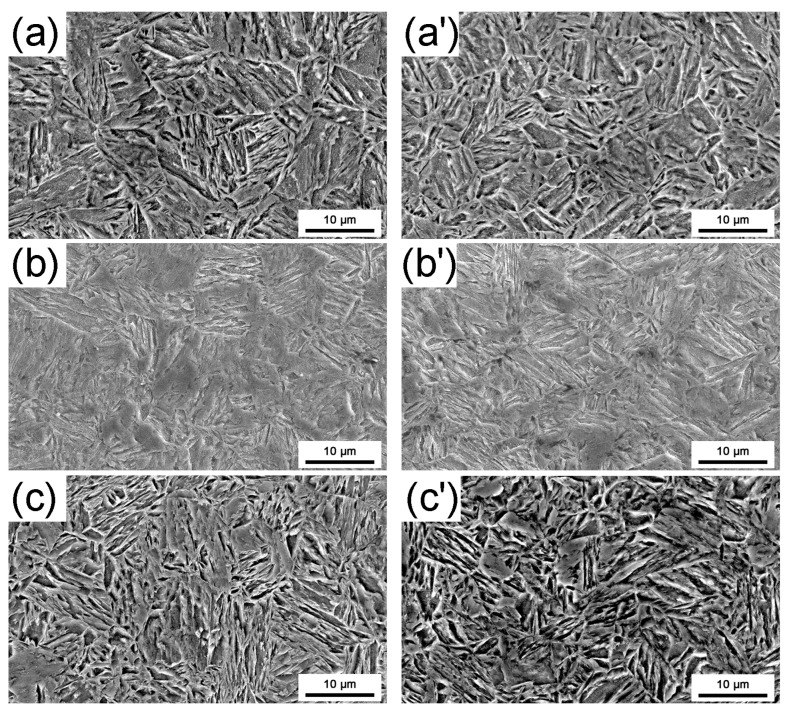
SEM images of marstenite quenching structure after heat treatment by the quenching oil of (**a**) traditional process and (**a’**) CSP process, the water of (**b**) traditional process and (**b’**) CSP process, and the 10 wt.% NaCl solution of (**c**) traditional process and (**c’**) CSP process.

SEM microstructure images for traditional and CSP processes following quenching are presented in Figure 3. Lath martensite can be obtained through quenching after the samples are treated at 920 °C for 4 min. The microstructure of steel quenched in the three quenching media is fully martensite. However, the martensitic lath size varies slightly depending on the cooling rate. In general, the faster the cooling rate, the finer the martensite lath after the phase transition. The martensite lath quenched in a 10 wt.% NaCl solution is the thinnest, while the martensite lath quenched in quenching oil is the thickest.

**Figure 4:**
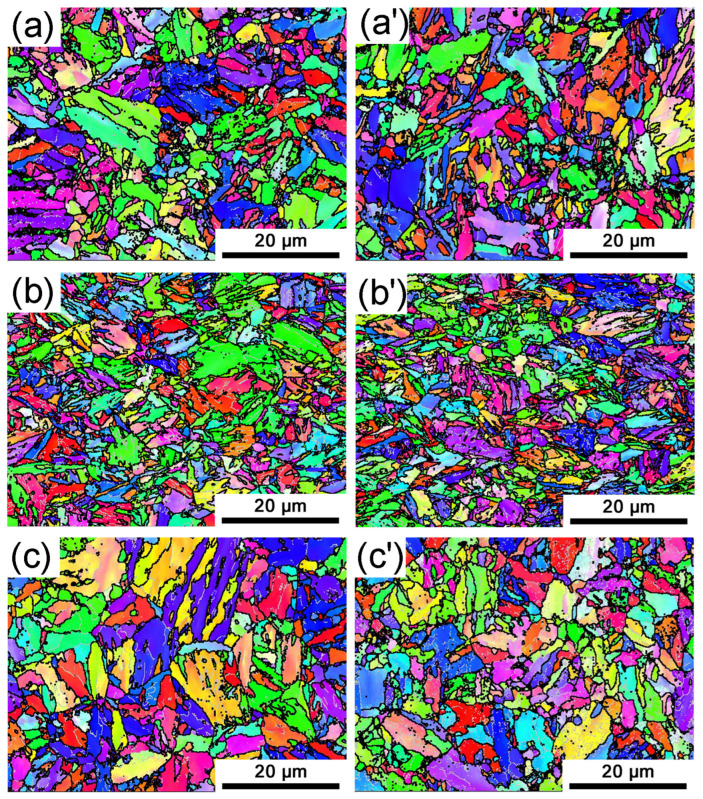
IPF maps of marstenite structure after quenched in the quenching oil of (**a**) traditional process and (**a’**) CSP process, the water of (**b**) traditional process and (**b’**) CSP process, and the 10 wt.% NaCl solution of (**c**) traditional process and (**c’**) CSP process.

EBSD characterization is shown in Figure 4. From the IPF images, the martensite blocks with low-angle boundaries can be clearly detected, and the martensite lath with similar orientations are distributed within the blocks. The martensite lath has straight boundaries, which are approximately parallel to the {111} plane of prior austenite, namely the habit plane. The four-level structure of martensite consists of prior austenite, martensite packet, martensite block, and martensite lath. A prior austenite grain is divided into three to five packets, which are collections of blocks with the same habit plane. Each block is composed of several laths with similar orientations. These laths are separated by retained austenite with a thickness of about 20 nm (unmeasurable by EBSD). These residual austenites contain a higher carbon content and are stable at room temperature.

**Figure 5:**
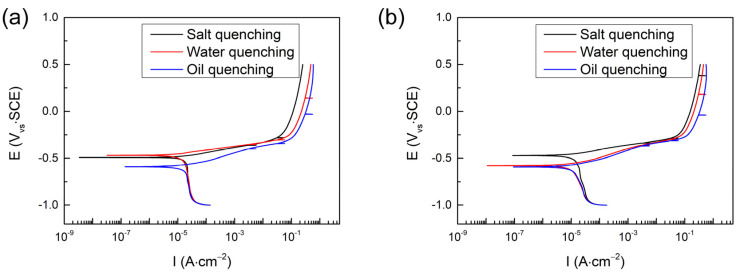
Potentiodynamic polarization curves for the samples quenched in the different quenching mediums in 3.5% NaCl solution: (**a**) traditional process, (**b**) CSP process.

Figure 5 shows the potentiodynamic polarization curves of all quenched samples by a different quenching medium. The cathodic branches of the potentiodynamic polarization curves indicate the process of cathodic oxygen reduction, and the anodic branches suggest the dissolution of anodic iron [34]. For hot-stamping steel produced by the traditional process, as seen in Figure 5a, the anodic branches reveal that all samples exhibit an analogous behavior with an active solution zone due to the nonpassivation of the electrode in a 3.5% NaCl solution. The anode current density of the oil-quenched sample is higher than that of the water-quenched sample, and higher than that of the 10% NaCl solution-quenched sample. In addition, a sudden change in the anodic branches means that there is a pitting breakdown occurring on the sample surface. For hot-stamping steel produced by the CSP process, as seen in Figure 5b, similar rules were found to the traditional process.

**Figure 6:**
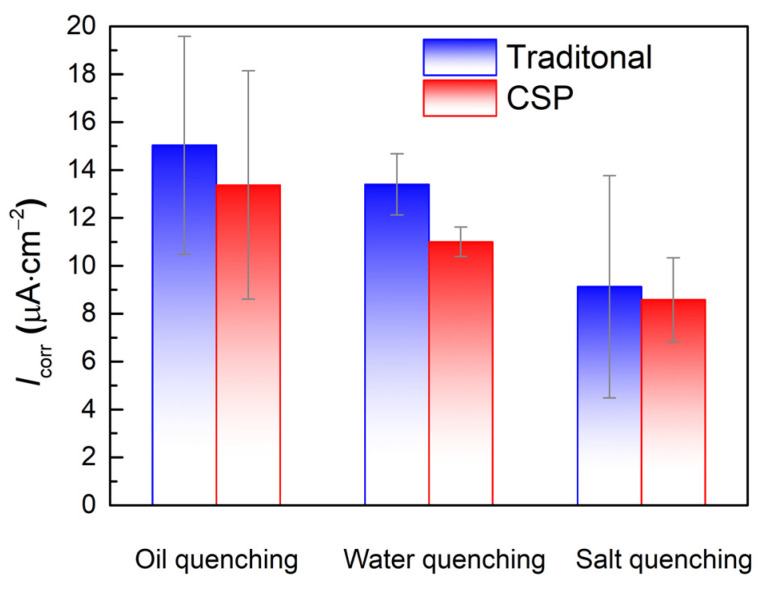
Calculated corrosion current density for the samples produced by traditional and CSP processes quenched in the different quenching mediums.

Tafel slope fitting was employed to obtain the corrosion current density and the results are shown in Figure 6. As the quenching cooling rate increases, the corrosion current density is 15 μA·cm^−2^, 13.4 μA·cm^−2^, and 9.1 μA·cm^−2^ for the traditional process, and is 13.4 μA·cm^−2^, 11 μA·cm^−2^, and 8.6 μA·cm^−2^ for CSP process. It can be seen that the quenching cooling rate will significantly affect the corrosion rate. The faster the cooling rate, the lower the corrosion rate. Furthermore, the corrosion current density of samples produced by the traditional process is higher than that of the CSP process at the same quenching cooling rate. 

**Figure 7:**
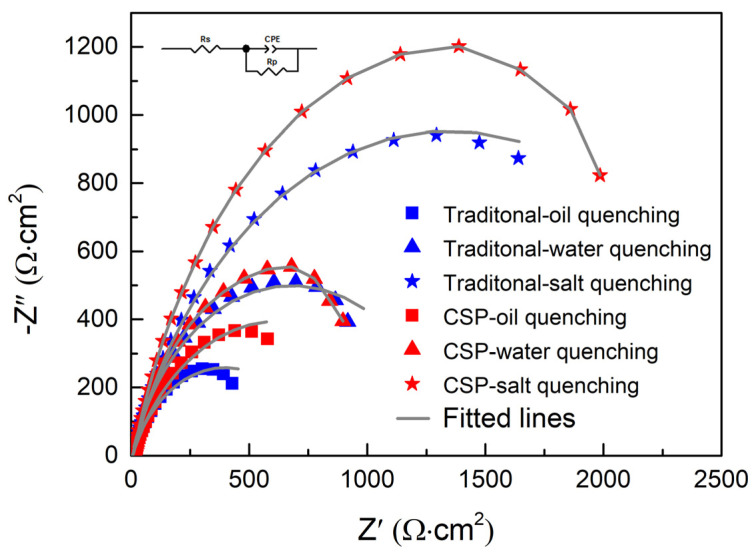
Electrochemical tests of Nyquist plots were tested for the samples produced by traditional and CSP processes quenched in the different quenching mediums.

Further electrochemical tests using EIS are shown in Figure 7. As the quenching cooling rate increases, the polarization resistance is 789 Ω·cm^2^, 1344 Ω·cm^2^, and 2537 Ω·cm^2^ for the traditional process, and is 1186 Ω·cm^2^, 1473 Ω·cm^2^, and 2887 Ω·cm^2^ for the CSP process. These results are in good agreement with the potentiodynamic polarization curves.

The corrosion rate is affected by the type of quenching medium used, primarily based on the fact that quenching speeds lead to a finer microstructure and smaller equivalent grain size. While one study [35] has indicated that grain boundaries, as crystal defects, can promote corrosion in metallic materials, others [36,37,38] have demonstrated that a finer grain size can improve corrosion resistance, and the grain boundaries can act as corrosion barriers to retard corrosion kinetics [39,40]. In this study, an increase in quenching speed resulted in finer martensite-equivalent grain size and lower corrosion rates, which would be consistent with the hypothesis that grain boundaries act as corrosion barriers.

**Figure 8:**
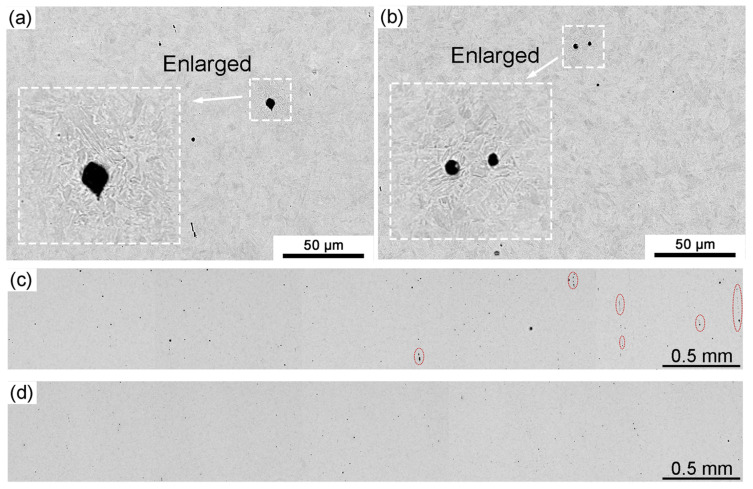
Matrix corrosion aggravated by the inclusions for the samples after quenching of (**a**) traditional process (**b**) CSP process, and the distribution of inclusions over a macro field of view for (**c**) traditional process (**d**) CSP process.

The presence of the second phase in the microstructure (mainly inclusions) would also affect the corrosion behavior due to their different structures from the matrix. Figure 8 shows the BSE images and the distribution of inclusions in steel produced by traditional and CSP processes, assisted by EDS analysis to classify the types of inclusions. In this study, Al_2_O_3_/Al inclusions and TiN inclusions were mainly considered. The results indicate that Al_2_O_3_/Al inclusions exhibit a spherical morphology and are generally larger in size, while the size of TiN inclusions is relatively small, and their morphology appears to be irregular. The detailed observations reveal that the Al_2_O_3_/Al inclusions can significantly accelerate the corrosion of the surrounding matrix, as indicated by the insets in Figure 8a,b. This is due to the difference in corrosion potentials between the inclusions and the matrix, which can lead to galvanic corrosion [41,42]. The more inclusions there are, the more severe the corrosion will be, theoretically. A wider view of the inclusions in the CSP process is shown in Figure 8c,d, where they are sparsely distributed and smaller in size.

**Figure 9:**
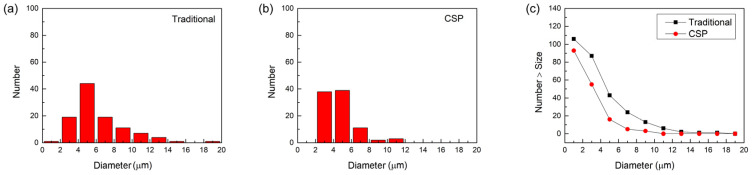
Statistics histograms of Al_2_O_3_/Al inclusions of (**a**) traditional process (**b**) CSP process, and (**c**) the cumulative distribution for the Al_2_O_3_/Al inclusions in two processes.

**Figure 10:**
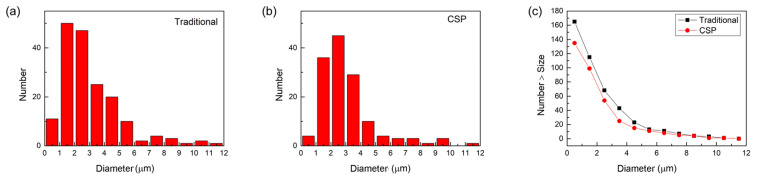
Statistics histograms of TiN inclusions of the (**a**) traditional process, (**b**) CSP process, and (**c**) the cumulative distribution for the Al_2_O_3_/Al inclusions in two processes.

The statistical analysis of inclusions in hot-stamping steel produced by the two processes is plotted in Figure 9 and Figure 10. The Al_2_O_3_/Al inclusions distribution in the two processes exhibits significant differences, while no significant difference is observed for TiN inclusions in this statistical method. For the traditional process, the average size of Al_2_O_3_/Al inclusions is 6.2 μm, while for the CSP process, it is 4.6 μm. The average size of TiN inclusions, on the other hand, was the same for both processes, at 3.1 μm. The average and maximum sizes of Al_2_O_3_/Al inclusions are both larger than those of TiN inclusions. Therefore, it is reasonable to believe that the difference in corrosion resistance between traditional and CSP processes is closely related to Al_2_O_3_/Al inclusions. The higher corrosion rate in the traditional process can be attributed to the presence of a large proportion of large-sized Al_2_O_3_/Al inclusions. Additionally, it can be observed from Figure 9c and Figure 10c that the total number of both Al_2_O_3_/Al and TiN inclusions in the traditional process is higher than that in the CSP process.

Due to the thin thickness of the billet (approximately 50–130 mm), the crystallizer in the CSP process is thin from the inlet to the outlet, and the casting nozzle is also thinner than in the conventional process. To prevent clogging and improve continuous casting, moderate calcium treatment is usually carried out after refining. The type of Al_2_O_3_/Al inclusions have some differences compared with traditional billets. The solidification time of the steel is shorter (1 min) and the cooling rate is faster (10^1^–10^2^ k/s) during CSP continuous casting [43,44]. The faster the solidification speed, the smaller the size of the inclusions. The larger cooling rate causes inclusions to have not enough time to generate and grow from the high-temperature phase, resulting in fewer large-sized inclusions in the CSP casting billet.

Inclusions in steel are unwanted impurities that are inevitably produced during the steelmaking process. Among the various types of inclusions, Al_2_O_3_ is the most common. Researchers have studied the impact of Al_2_O_3_ inclusions on the localized corrosion of steel. It has been observed that the presence of Al_2_O_3_-enriched inclusions in the steel can lead to the preferential dissolution of the surrounding steel through galvanic coupling [45]. Furthermore, the interface between the Al_2_O_3_ inclusions and the steel matrix has been found to be a site of pitting corrosion [46,47]. Therefore, it is clear that Al_2_O_3_ inclusions play a crucial role in the localized corrosion of steel. The CSP process, which produces steel with fewer and smaller inclusions, results in steel with better corrosion resistance compared to the traditional process. This is due to a reduced number of inclusions and their smaller size, resulting in fewer galvanic couples and, therefore, less localized corrosion occurring.

## 4. Conclusions

The microstructure and mechanical properties, and especially the corrosion behavior, were investigated between the traditional and CSP processes. The following conclusions were drawn.

(1) The original microstructure of hot-stamping steel produced by the traditional process and the CSP process is different. After quenching, the microstructures transform into full martensite, and their mechanical properties meet the 1500 MPa grade. The faster the quenching speed, the higher the strength;
(2) Corrosion tests showed that different quenching speeds can affect the corrosion rate, i.e., the faster the quenching rate, the finer the microstructure, and the smaller the corrosion rate of the steels. The corrosion resistance of hot-stamping steel produced by the CSP process is slightly better than that of traditional processes;
(3) The effect of quenching speeds on the corrosion rate is based on the hypothesis that a finer grain size can improve corrosion resistance, and the grain boundaries can act as corrosion barriers to retard corrosion kinetics. The CSP process, which produces fewer and smaller inclusions, results in better corrosion resistance due to the reduced number of inclusions and their smaller size with fewer galvanic couples.

## Figures and Tables

**Table 1:** Chemical composition of 1500 MPa grade hot-stamping steel after traditional and CSP processes (wt.%).

Steels	C	Si	Mn	Al	Cr	Ti	B	Fe
CSP	0.23	0.28	1.15	0.029	0.21	0.04	0.0028	Bal.
Traditional	0.26	0.31	1.24	0.029	0.19	0.038	0.0025	Bal.

**Table 2:** Mechanical property parameters of hot-stamping steel produced by traditional and CSP processes after quenching include yield strength (σ_s_), tensile strength (σ_b_), and total elongation (E_total_).

Steel		σ_y_/MPa	σ_UTS_/MPa	Elongation/%
Quenching oil	Traditional	1016 ± 13	1469 ± 3	7.0 ± 0.6
	CSP	986 ± 17	1423 ± 13	7.8 ± 0.8
Water	Traditional	1203 ± 29	1674 ± 17	6.8 ± 0.8
	CSP	1194 ± 31	1592 ± 25	6.8 ± 0.7
10 wt.% NaCl	Traditional	1187 ± 13	1738 ± 21	5.9 ± 0.3
	CSP	1234 ± 48	1693 ± 16	7.7 ± 0.9

## Data Availability

Not applicable.
